# Tandem Specialty Management of Complex Lower Extremity Wounds: A Report of Three Cases

**DOI:** 10.7759/cureus.6193

**Published:** 2019-11-19

**Authors:** Adam Carr, Joseph Long, Ralph J Napolitano, Nicholas A Cheney, Jeffrey Gittins

**Affiliations:** 1 Orthopedic Surgery, McLaren Greater Lansing, Lansing, USA; 2 Medicine, Ohio State University, Columbus, USA; 3 Podiatry and Wound Care, OrthoNeuro, Columbus, USA; 4 Foot and Ankle Surgery, OrthoNeuro, Columbus, USA

**Keywords:** complex wounds, lower extremity wounds, tandem specialty, diabetic ulcers, podiatry, orthopedic surgery, team approach, gastrocnemius recession

## Abstract

Complex lower extremity wounds present a unique problem to foot and ankle clinicians, with many obstacles to achieving a successful outcome. The decreased vasculature of the lower extremities creates environments where wounds lack the resources to properly heal on their own. Conditions such as diabetes mellitus and smoking can exacerbate these issues by further decreasing vascular flow providing resources to the wound. For physicians trained in orthopedic foot and ankle surgery, they often do not receive training in advanced wound care, whereas podiatric surgeons can obtain fellowship training in wound care management. This dynamic presents a unique opportunity for tandem management of complex lower extremity wounds, which can decrease patient morbidity and the costs associated with care. We present three cases of complex wounds managed in a tandem fashion that achieved optimal outcomes after both orthopedic surgery and podiatric surgery were involved. These cases illustrate the potential benefits associated with tandem wound management in foot and ankle surgery,

## Introduction

In patients with a decrease in blood circulation, wounds to the lower extremities can become difficult to properly heal [1]. These wounds often arise due to complications in diseases such as Charcot-Marie-Tooth, diabetes mellitus, and rheumatoid arthritis or after an invasive surgical procedure [2-3]. Wound healing presents a serious issue due to the increased risk of infection when the skin barrier is disrupted for a prolonged period of time. An infection could produce many adverse effects in the area, including deep bone infections, which may require amputation [4].

Patients exhibiting diseases with peripheral neuropathy are at risk for ulcers forming on the bottom of the foot [3,5]. These ulcers can go unnoticed for extended periods of time, allowing them to progress or become infected. One cause of these ulcers is intense pressure on the skin [3]. In cases where complex ulcers form under the forefoot, a gastrocnemius recession can provide an offloading of weight, to allow the wound to properly heal [5].

After extensive midfoot or forefoot reconstruction, patients with poor circulation, secondary to disease, can have difficulty with wound healing. These patients may need extraordinary measures to begin the healing process or an extended period of time for the wound to properly heal.

Orthopedic surgeons trained in foot and ankle surgery and podiatric surgeons trained in wound care can work together to provide resources and appropriate care for complicated wounds. Therefore, a tandem management approach using both specialties provides patients with the appropriate treatment plan to prevent further complications during their recovery.

## Case presentation

Case 1

A 75-year-old female presented to her orthopedic surgeon in May 2015 with a complaint of bilateral foot pain at the great toe joint. The patient had undergone previous surgeries to correct the pain but all were unsuccessful. After the consultation, a left gastrocnemius recession and a left midfoot reconstruction were performed to offload excess weight through the ball of the foot.

**Figure 1:**
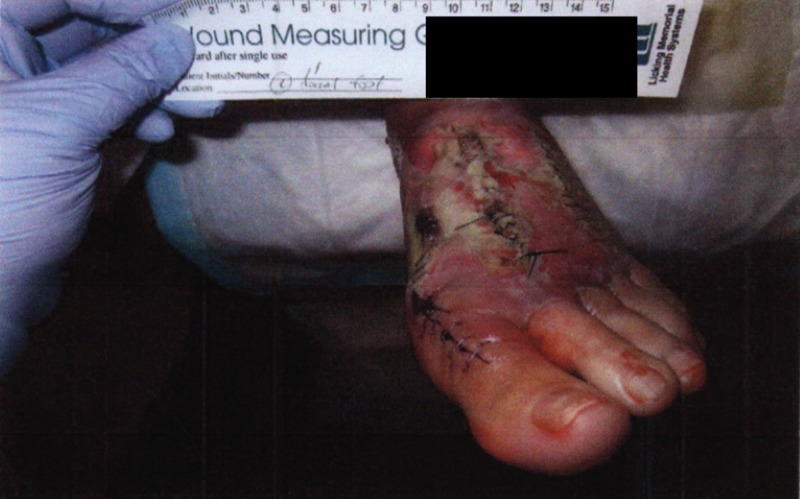
Gross image of initial presentation showing swelling blisters and wound dehiscence

**Figure 2:**
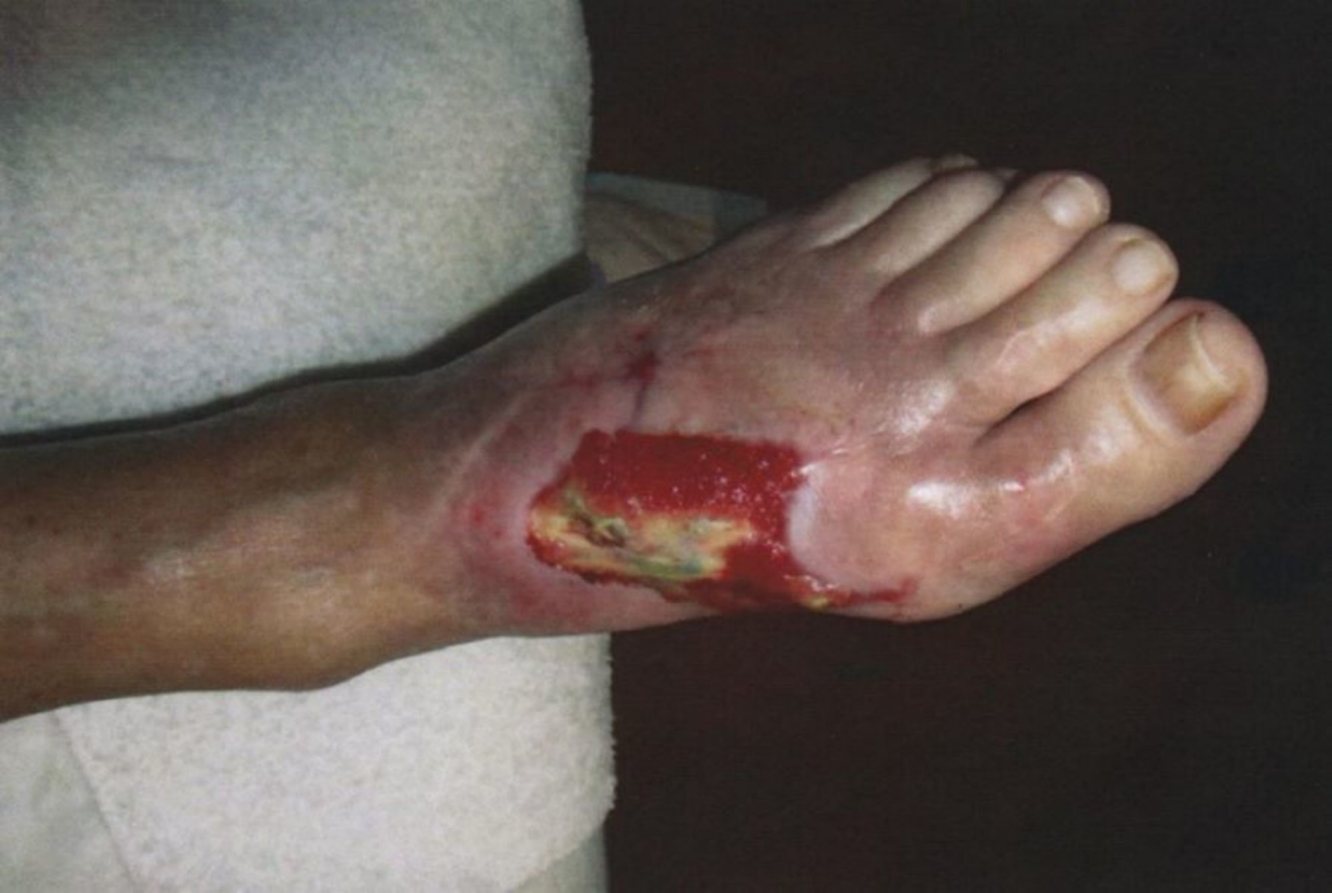
Gross image of progress at three weeks showing healthy granular tissue after necrotic tissue removal

**Figure 3:**
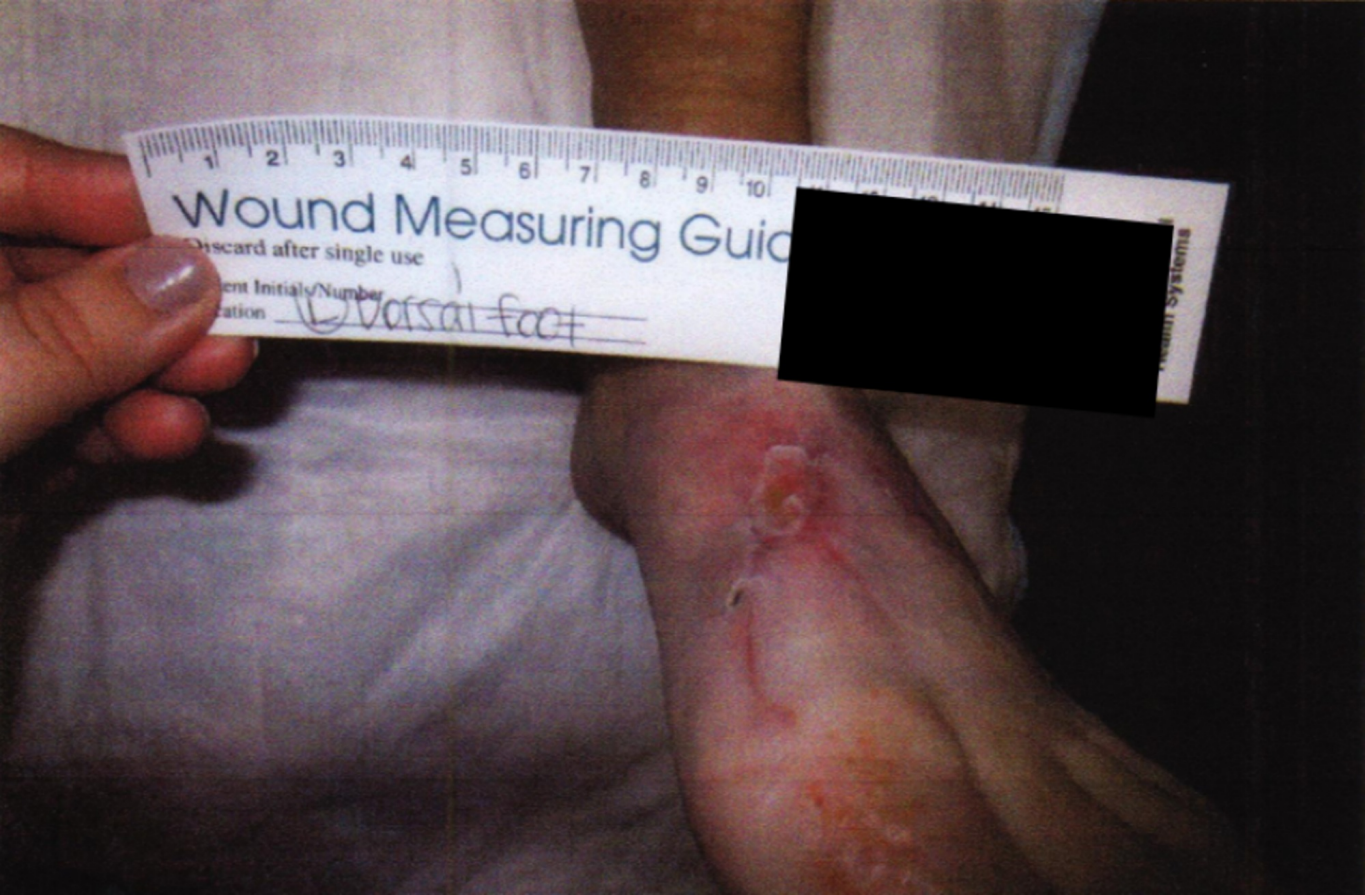
Gross image of progress at six months showing continued healing of the dorsal wound

At the postoperative appointment one week after surgery, the skin around the incision appeared to be ecchymotic without signs of breakdown or infection. Two weeks postoperatively, there were large blisters with wound dehiscence. The skin appeared broken down, although the incision was well-approximated. At this time, the patient was referred to a podiatric surgeon with advanced wound training by her orthopedic surgeon (Figures 1-3).

The wound care protocol for this patient used Adaptic dressing (Johnson and Johnson, NJ, US) along the staple lines together with Aquacel silver (ConvaTec Group PLC, Deeside, United Kingdom) and a non-bordered film dressing to be changed every other day or as needed with strict non-weight bearing. Five weeks postoperatively, necrotic tissue was removed and healthy granular tissue was appreciated underneath as well as subtle fibrinogen. In the next visit, during postoperative week six, negative pressure therapy began and was continued for one month. Ten weeks postoperatively, silver nitrate was placed on some of the hypertrophic areas that remained. Eleven weeks postoperatively, a skin graft was placed over the remaining wound and curettage was performed to debulk some of the remaining slightly thick hypertrophic tissue. Over the course of the next three months, the wound was cared for by the certified wound specialist. Debridement and cleaning the wound happened on a weekly basis until the wound healed and the patient was discharged from care.

Case 2

**Figure 4:**
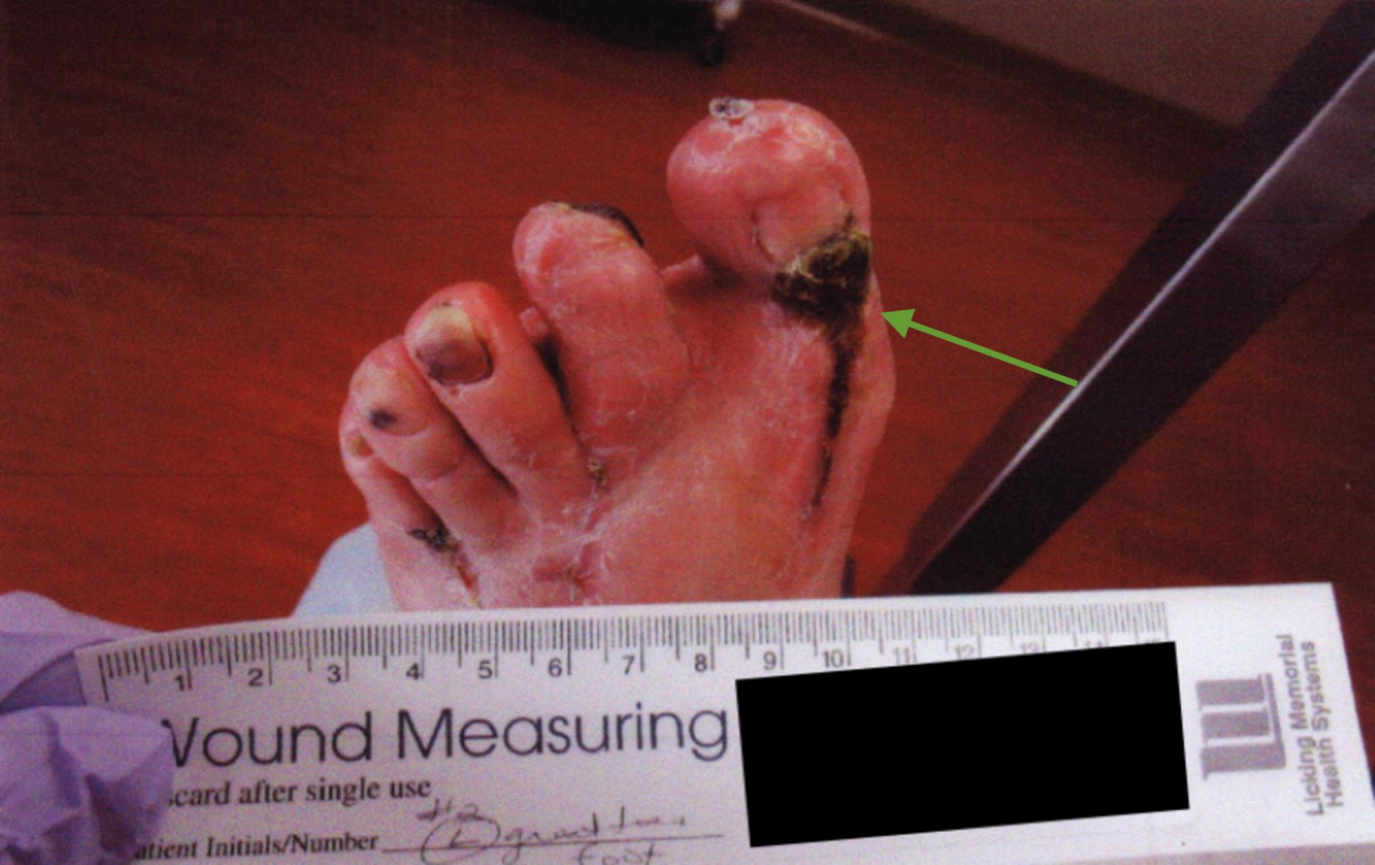
Gross image of the initial presentation showing eschar on the dorsal left foot incision

**Figure 5:**
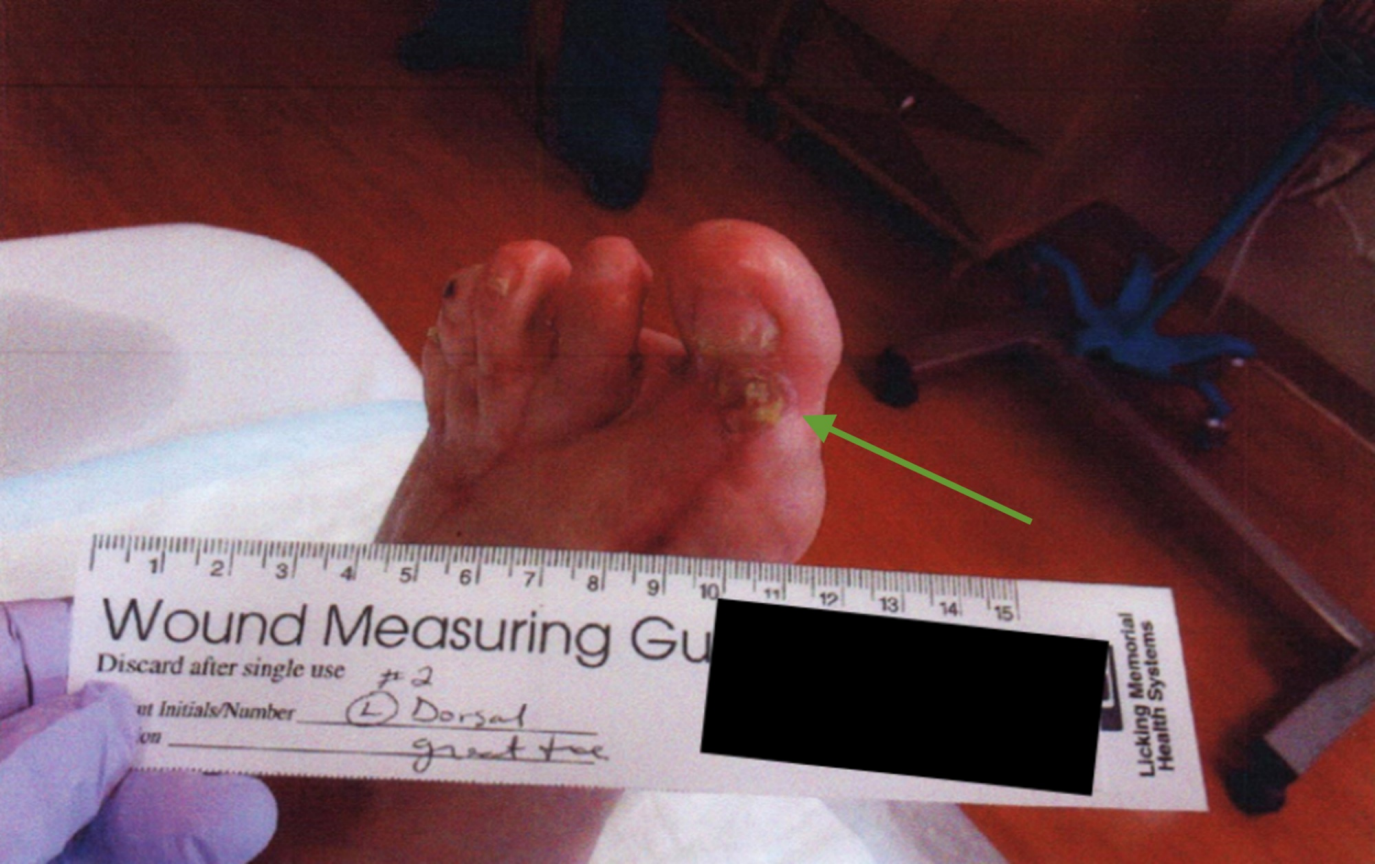
Gross image of progress at two months showing diminished eschar

**Figure 6:**
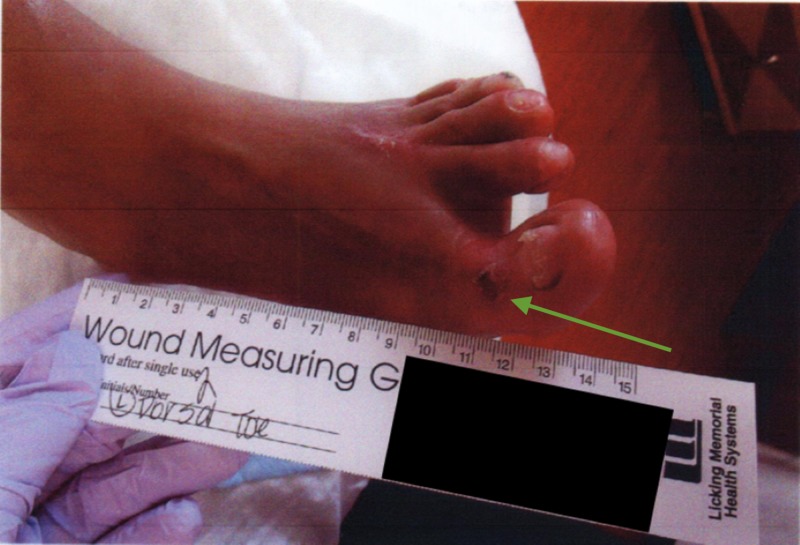
Gross image of progress at three months showing complete epithelialization

A 64-year-old man with a past medical history of rheumatoid arthritis underwent an extensive forefoot reconstruction of his left lower extremity in January 2015 at the hands of his orthopedic surgeon. The immediate postoperative course was uneventful, but he gradually developed wound-healing issues. The patient was referred to a podiatric surgeon with advanced wound training in February 2015 with eschar on the dorsal left foot incision and a second toe distal eschar with some fibrinogen and suggestion of granular tissue. The patient was previously shown to have adequate perfusion in the extremity via noninvasive arterial studies (Figures 4-6).

Initially, the patient was treated with clean dressings utilizing AMD gauze (Cardinal Health, Ohio, US), changed as needed. He was also instructed to continue elevating the leg above heart level as much as possible. Multiple surgical debridements were performed between late February and early March 2015. Treatment following debridement consisted of antibiotic-loaded bone cement pellets, daily dressing changes using gentamicin cream, and gauze for the second toe; Aquacel silver with saline covered by gauze for the great toe was changed every two or three days in addition to oral antibiotics. Follow-up appointments took place weekly through March. All wounds were completely epithelialized by May 2015, with some tissue loss. The patient was discharged and advised to keep the area clean and dry, check it daily, and follow up with his orthopedic surgeon.

Case 3

**Figure 7:**
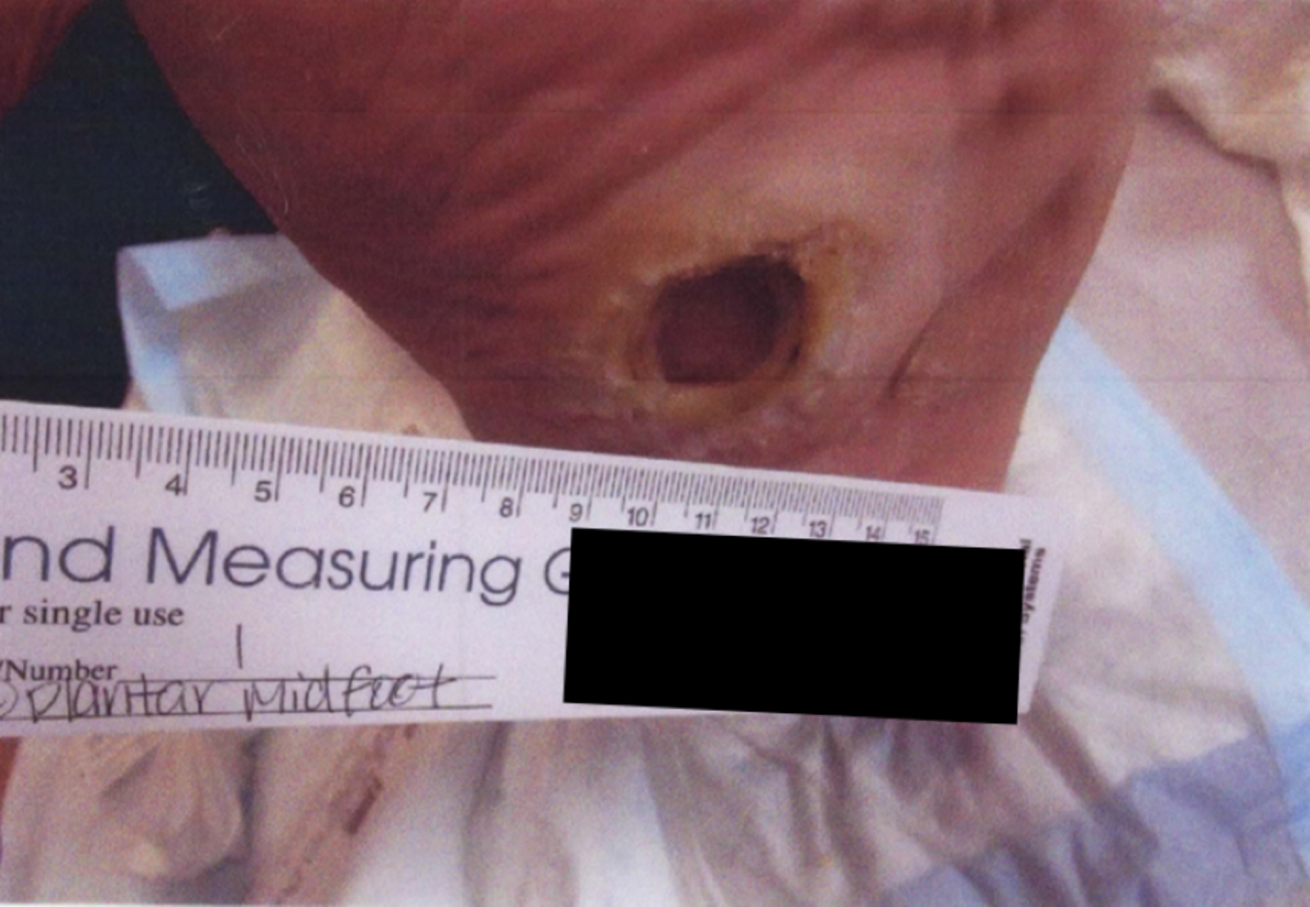
Gross image showing 6 cm left foot plantar ulceration

**Figure 8:**
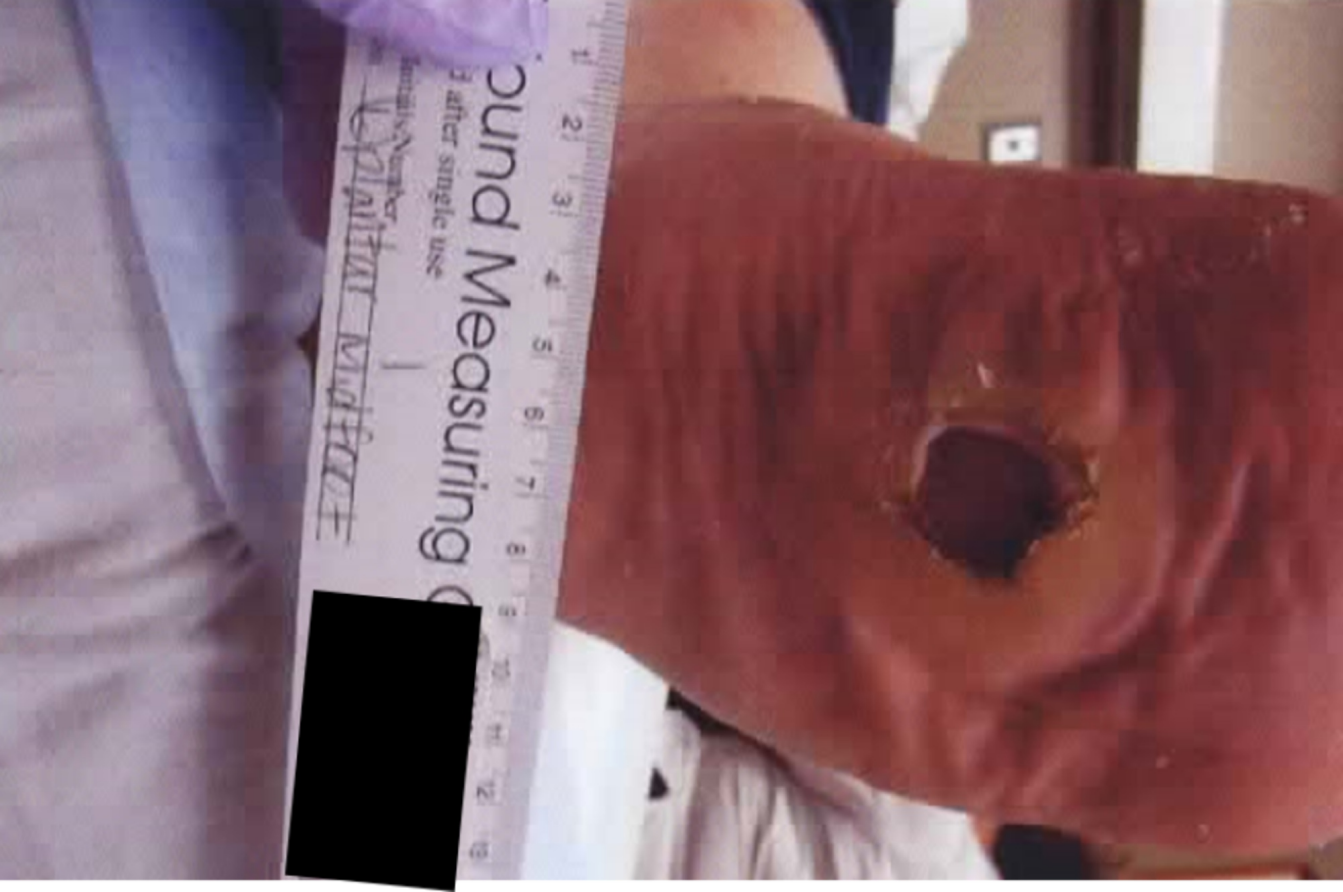
Gross image of progress at three months showing minimal decrease in ulcer size before gastrocnemius recession

**Figure 9:**
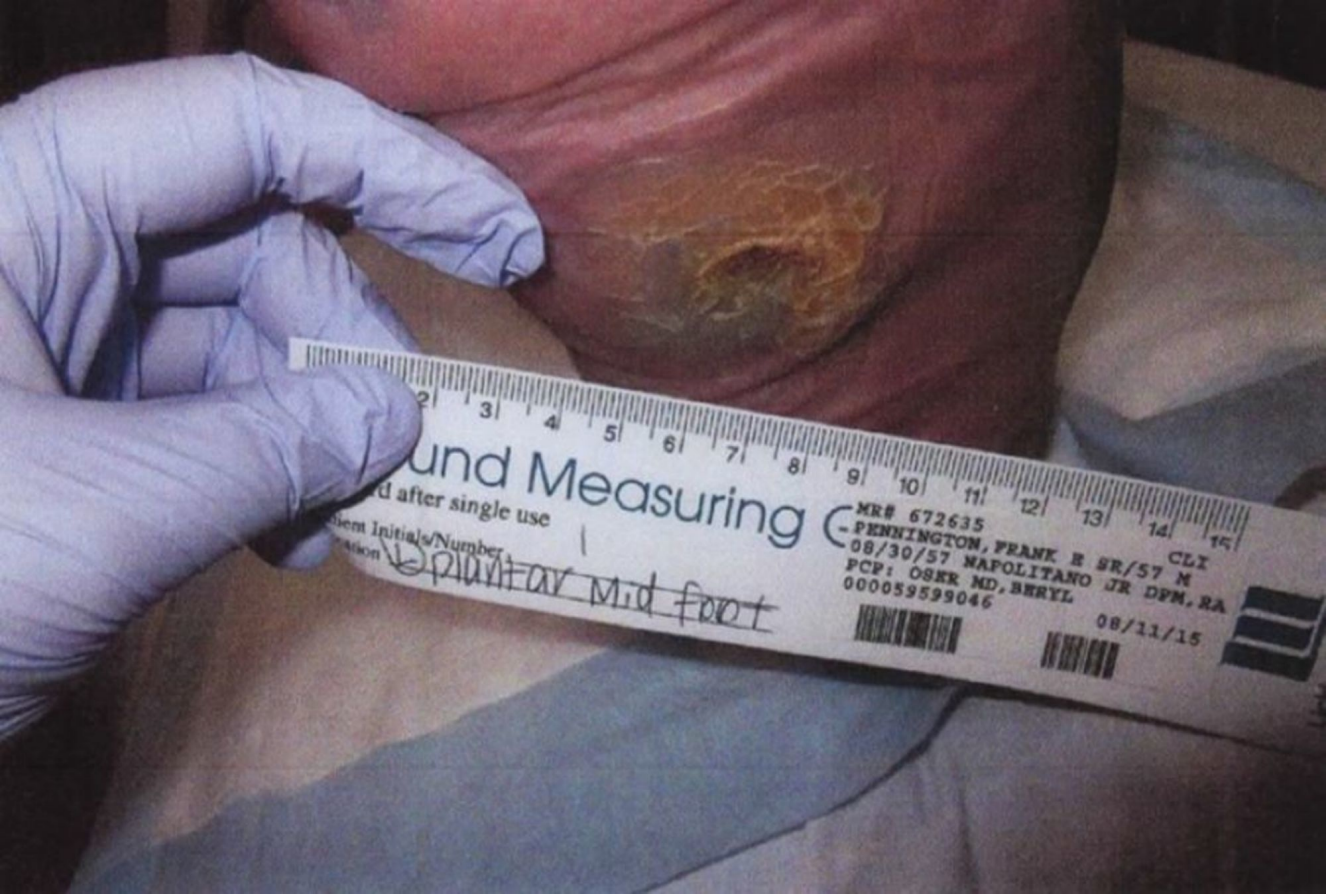
Gross image of progress two months after the tandem operation showing significant improvement in healing

A 58-year-old man with a history of diabetic foot disease and Charcot-Marie-Tooth disease presented to the wound clinic in August 2014 for the further management and surveillance of a left foot plantar ulceration. At this time, the wound was approximately 6 cm in diameter and the large, granular deep defect was without significant periwound hyperkeratosis. He experienced episodes of improvement and worsening throughout the course of this wound. Perfusion was noted and obvious neuropathy existed (Figures 7-9).

The wound was treated with dressing changes every other day using AMD gauze covered with Kerlix roll (Cardinal Health). He was also instructed to elevate his legs and to wear an offloading shoe with SpandaGrip (Meditech International, FL, US). A Crowe boot (Crowe Boot & Shoe, IL, US) was also prescribed. Debridement took place in September 2014, in which periwound hyperkeratosis was taken down. In November 2014, augmentin was prescribed to prevent infection and CelluTome skin grafting (Acelity, Texas, US) was offered. The patient wished to proceed with the skin grafting and the appropriate procedure was performed. After the procedure, wound dressing was applied solely at the wound clinic, and the patient was instructed to leave dressings on between visits, with follow-up appointments every three to four days through mid-December. In mid-December, the ulcer was continuing to improve; it was now approximately 3 cm in diameter. Wound care was allowed to be transferred back to the patient’s responsibility, with instructions to pack with Aquacel silver and wrap with gauze. Negative pressure therapy was suggested in February 2015 due to delayed wound healing and was begun with a wound vacuum at that time.

By mid-March 2015, a plateau phase had been reached in the patient’s wound healing. It was at this point that a prophylactic surgery was discussed. The patient was a prime candidate for this procedure because his wound was no longer making significant gains, but he had shown the capability to heal. The patient had also developed several ulcerations on the second and third toes of his right foot due to hammertoe contractures. It was suggested to the patient that hammertoe correction with distal phalangectomy will help offload the distal aspect of the right toes. At the same time, it was recommended that a gastrocnemius recession be performed on the left leg to help further offload the patient’s forefoot. The patient agreed to both surgical interventions.

The patient requested the surgery be delayed until June 2015. Wound management continued with Aquacel silver wrapped with gauze and the wound vacuum, up to the day of the procedure. The surgery was performed in a tandem fashion with both an orthopedic surgeon and a wound specialist performing specific interventions. The patient’s equinus contracture and hammertoe deformities were successfully corrected surgically, with no intraoperative complications. The patient developed some stasis ulcerations and toe infections postoperatively, which were managed with compression therapy, Coban 2 (3M Health Care, Minnesota, US), and Keflex. Surgical intervention was successful in continuing the progression of the overall healing process of his left foot plantar ulceration. By mid-August 2015, the plantar ulceration, hammertoe, and gastrocnemius recession incisions were completely healed and the stasis ulceration was improving. The patient was placed into a walking boot to prevent further breakdown until his diabetic shoes were available.

## Discussion

The complex nature of chronic wound management provides an opportunity for implementing a tandem specialty approach. Two specialties that have significant potential for improving outcomes in patients battling complex lower extremity wounds are podiatric surgeons with advanced wound training and orthopedic foot and ankle surgeons.

Lower extremity wounds are exceedingly common in patients with comorbidities such as diabetes mellitus and Charcot-Marie-Tooth disease, with some prevalence estimates approaching nearly 25% [6]. The effective management of these wounds can ultimately be the difference between saving or losing a limb [7]. The literature contains multiple examples of the interdisciplinary management of foot and ankle wounds [7-11], however, we were unable to find any examples in PubMed of wound cases being managed exclusively between wound specialists, such as a podiatric surgeon with advanced wound training, and orthopedic surgeons. The three cases presented in the current case report are a few examples of what can be expected when patients are efficiently managed within separate, but related, specialties to achieve the most desirable outcome.

The most promising feature of the system implemented for these patients is its bidirectional nature. One direction is best illustrated by patients who developed surgical dehiscence or significantly delayed postoperative healing (Cases 1-2). Orthopedic surgeons operating on the foot and ankle are capable of handling many wound complications. However, understanding that referring the patients who develop major complications to wound specialists who are experts in the management of complex wounds was the catalyst these patients needed in order to overcome their wounds. The second direction is demonstrated by the wound specialist requesting assistance from the orthopedic surgeon to perform surgical interventions as part of the wound treatment protocol. Case 3 illustrates a patient obviously suffering from forefoot overloading. There was little likelihood of this patient healing the wound permanently without finding a way to offload the forefoot region. Performing a gastrocnemius recession was a vital step in healing the wound.

## Conclusions

With complex lower extremity wounds, patient morbidity is greatly affected, and the high risk of further complications makes management an urgent concern. In this study, tandem management between orthopedic and podiatric surgeons handling complex lower extremity wounds resulted in favorable outcomes. This case series serves as an example of how the interprofessional management of complex medical problems can lead to better outcomes for patients in foot and ankle surgery.

## References

[1] Folk JW, Starr AJ, Early JS (1999). Early wound complications of operative treatment of calcaneus fractures: analysis of 190 fractures. J Orthop Trauma.

[2] Forsythe RO, Hinchliffe RJ (2015). Assessment of foot perfusion in patients with a diabetic foot ulcer. Diabetes Metab Res Rev.

[3] Lew DP, Waldvogel FA (1997). Osteomyelitis. N Engl J Med.

[4] Reiber GE, Vileikyte L, Boyko EJ, del Aguila M, Smith DG, Lavery LA, Boulton AJ (1999). Causal pathways for incident lower extremity ulcers in patients with diabetes from two settings. Diabetes Care.

[5] Cychosz CC, Phisitkul P, Belatti DA, Glazebrook MA, DiGiovanni CW (2015). Gastrocnemius recession for foot and ankle conditions in adults: evidence based recommendations. Foot Ankle Surg.

[6] Sumpio BE (2000). Foot ulcers. New Eng J Med.

[7] Rogers LC, Andros G, Caporusso J, Harkless LB, Mills JL Sr, Armstrong DG (2010). Toe and flow: essential components and structure of the amputation prevention team. J Am Podiatr Med Assoc.

[8] Hingorani A, LaMuraglia GM, Henke P (2016). The management of diabetic foot: a clinical practice guideline by the Society for Vascular Surgery in collaboration with the American Podiatric Medical Association and the Society for Vascular Medicine. J Vasc Surg.

[9] Armstrong DG, Bharara M, White M (2012). The impact and outcomes of establishing an integrated interdisciplinary surgical team to care for the diabetic foot. Diabetes Metab Res Rev.

[10] Sumpio BE, Armstrong DG, Lavery LA, Andros G (2010). The role of interdisciplinary team approach in the management of the diabetic foot: a joint statement from the Society for Vascular Surgery and the American Podiatric Medical Association. J Am Podiatr Med Assoc.

[11] Cho EH, Garcia R, Pien I, Thomas S, Levin LS, Hollenbeck ST (2014). An algorithmic approach for managing orthopaedic surgical wounds of the foot and ankle. Clin Orthop Relat Res.

